# Increased Excretion of C4-Carnitine Species after a Therapeutic Acetylsalicylic Acid Dose: Evidence for an Inhibitory Effect on Short-Chain Fatty Acid Metabolism

**DOI:** 10.5402/2011/851870

**Published:** 2011-02-22

**Authors:** Catharina M. C. Mels, Peet Jansen van Rensburg, Francois H. van der Westhuizen, Pieter J. Pretorius, Elardus Erasmus

**Affiliations:** Centre for Human Metabonomics, North-West University (Potchefstroom Campus), Private Bag X6001, Potchefstroom 2522, South Africa

## Abstract

Acetylsalicylic acid and/or its metabolites are implicated to have various effects on metabolism and, especially, on mitochondrial function. These effects include both inhibitory and stimulatory effects. We investigated the effect of both combined and separate oral acetylsalicylic acid and acetaminophen administration at therapeutic doses on the urinary metabolite profile of human subjects. In this paper, we provided *in vivo* evidence, in human subjects, of a statistically significant increase in isobutyrylcarnitine after the administration of a therapeutic dose of acetylsalicylic acid. We, therefore, propose an inhibitory effect of acetylsalicylic acid on the short-chain fatty acid metabolism, possibly at the level of isobutyryl-CoA dehydrogenase.

## 1. Introduction

**Figure 1:**
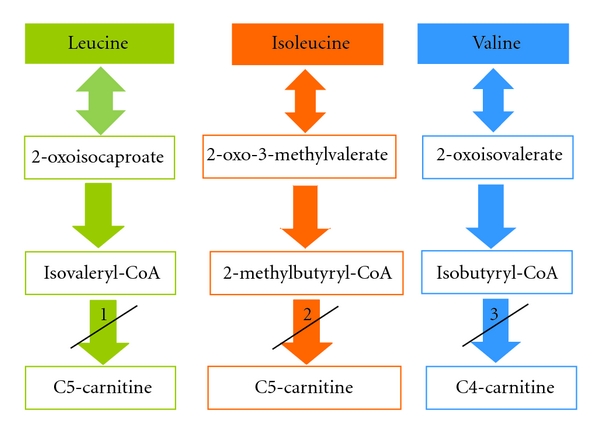
Proposed inhibition by salicylic acid of the branched-chain amino acid catabolism. Inhibition (or deficiency) of (1) isovaleryl-CoA dehydrogenase, (2) 2-methylbutyryl-CoA dehydrogenase, and (3) isobutyryl-CoA dehydrogenase, will result in the accumulation of C4-carnitine and C5-carnitine species.

The dehydrogenation of acyl-CoA intermediates in the catabolism of fatty acids and branched-chain amino acids in humans is catalysed by the mitochondrial acyl-CoA dehydrogenase enzymes [1, 2]. Several inherited defects in this group of enzymes have been characterised. Defects in, or inhibition of, isovaleryl-CoA dehydrogenase and 2-methylbutyryl-CoA dehydrogenase result in the accumulation of C5-carnitine species [3] whilst defects in, or inhibition of, short-chain acyl-CoA dehydrogenase and isobutyryl-CoA dehydrogenase result in the accumulation of C4-carnitine species [2] (Figure 1).

In the human body, acetylsalicylic acid is hydrolysed to salicylic acid, which is excreted in its conjugated form with glycine and glucuronic acid, in its hydroxylated form as 2,5-dihydroxybenzoic acid, or unchanged as salicylic acid [4]. Numerous *in vitro* and *in vivo* studies have been conducted that demonstrated various effects of acetylsalicylic acid (and its metabolites) on the metabolism and, especially, on mitochondrial function, such as the uncoupling of oxidative phosphorylation [5, 6], inhibition of fatty acid oxidation [5–8] with the concomitant stimulation of cytochrome P450 dependent *ω*-oxidation [9], and, therefore, accumulation of dicarboxylic acids [10]. In addition, the inhibition of both the Krebs cycle enzymes *α*-ketoglutarate dehydrogenase, and succinate dehydrogenase [6, 11], as well as an increased flux through the Krebs cycle [12], have been reported. Other effects of salicylic acid include the stimulation of oxygen consumption, stimulation of ATP hydrolysis [5], the activation of pyruvate dehydrogenase, and the inhibition of gluconeogenesis [12]. It can also lead to decreased blood glucose concentrations, increased hepatic triglycerides [6], and a slight increase in (iso)butyryl-CoA, *β*-methylcrotonyl-CoA, isovaleryl-CoA, and octanoyl-CoA concentrations [13].

The exact mechanism of inhibition that acetylsalicylic acid and/or its metabolites exert on fatty acid oxidation is not clear, since different studies indicate different sites of inhibition. These include an inhibitory effect on the activation and transportation of medium- and long-chain fatty acids into the mitochondria [7], due to the sequestration of extramitochondrial coenzyme A and carnitine [6], inhibition of the medium-chain acyl-CoA synthetase [8], or inhibition of the carnitine acyltransferase (CAT) enzymes [14]. Conversely, it was demonstrated that the target of inhibition was found to be at the level of the long-chain 3-hydroxyacyl-CoA dehydrogenase (LCHAD) activity of the mitochondrial trifunctional *β*-oxidation enzyme (MTE) and not at the level of uptake or activation of fatty acids [15].

While performing biotransformation metabolism and oxidative stress status profiling studies, on individuals referred to our laboratory, by using acetylsalicylic acid and acetaminophen as probe substrates, we made the rather startling observation of the presence of elevated C3-, C4-, and C5-carnitine species in their urine. These observations led to a more in-depth investigation into the nature of these acylcarnitine species and whether their increased excretion was due to the separate or combined effect of the acetylsalicylic acid and acetaminophen administration. Since the accumulation of C4- and C5-carnitine species is predominantly associated with deficient branched-chain amino acid metabolism at the level of different acyl-CoA dehydrogenase enzymes [2, 3], we hypothesized that acetylsalicylic acid administration is associated with the elevation of these species, which may be linked to the inhibition of the metabolism of C4 and C5 fatty acids (Figure 1). The formulation of this hypothesis was supported by the previous observation that a slight increase in (iso)butyryl-CoA, *β*-methylcrotonyl-CoA, isovaleryl-CoA, and octanoyl-CoA concentrations occur after acetylsalicylic acid intake [13].

This investigation provides the first evidence of a significant increase in isobutyrylcarnitine excretion following acetylsalicylic acid intake at therapeutic doses in humans, and we propose that this accumulation may be due to the inhibitory effect of acetylsalicylic on the metabolism of C4 and C5 fatty acids.

## 2. Materials and Methods

### 2.1. Subjects

The test subjects were divided into two groups. The first group included 30 test subjects, 19 female and 11 male between the ages of 12 and 65 years. Participants in this group were originally referred for biotransformation metabolism and oxidative stress status assessment, which include the administration of both acetaminophen and acetylsalicylic acid. The second group included seven randomly chosen participants from the first group and were used to ascertain whether the observed effects were due to acetylsalicylic acid intake, acetaminophen intake, or a combination thereof. The study adhered to the guidelines set in the Declaration of Helsinki. Approval for this work was obtained from the Ethics Committee of the North-West University, and informed consent was obtained from all participating subjects.

### 2.2. Loading Protocol and Sample Collection

Fasting baseline urine samples of all the test subjects in the first test group were collected the morning of the test day. At 21:00, on the same day, all the test subjects ceased eating and drinking (except water), emptied their bladders, and took therapeutic doses (1000 mg) acetaminophen and (600 mg) acetylsalicylic acid, as recommended by the different pharmaceutical companies for the relieve of mild to moderate pain. The total volume of urine excreted for ten hours after administration of acetaminophen and acetylsalicylic acid was collected and the quantity of urine excreted was documented. 

The second test group followed a protocol to determine if the observed effect on the acylcarnitine profile was due to acetylsalicylic acid intake, acetaminophen intake, or a combination thereof. On the first day at 21:00, all subjects ceased eating and drinking (except water) for the day and collected a baseline urine sample, after which they emptied their bladders. All subjects took 600 mg of acetylsalicylic acid and collected all the overnight urine until 7:00 the next morning. On the second occasion, the same protocol was followed except that all the subjects took 1000 mg of acetaminophen and collected all the overnight urine until 7:00 the next morning and on the third occasion all the subjects took both 600 mg acetylsalicylic acid and 1000 mg acetaminophen and collected all the overnight urine until 7:00 the next morning. Subjects waited for a minimum of three days before going on to the next protocol.

### 2.3. Reagents

The following reagents were purchased from Merck Chemical Co. (Darmstadt, Germany) acetonitrile, formic acid, and methanol. 3N butanolic HCl, valine, leucine, isoleucine, phenylalanine, methionine, citrulline, glycine, and lysine were purchased from Sigma-Aldrich Co. (St. Louis, USA). The following carnitine and acylcarnitine standards and deuterated carnitine and acylcarnitine standards were obtained from Dr. H. J. ten Brink, Free University Hospital (Amsterdam,  The Netherlands):  L-carnitine·HCl, acetyl-L-carnitine·HCl, propionyl-L-carnitine·HCl, isovaleryl-L-carnitine·HCl, octanoyl-L-carnitine·HCl, hexadecanoyl-L-carnitine·HCl, [methyl-d_3_]-L-carnitine·HCl, [d_3_]-acetyl-L-carnitine·HCl, [3,3,3-d_3_]-propionyl-L-carnitine·HCl, [d_9_]-isovaleryl-L-carnitine·HCl, [8,8,8-d_3_]-octanoyl-L-carnitine·HCl, and [16,16,16-d_3_]-hexadecanoyl-L-carnitine·HCl. The following deuterated amino acids were obtained from Cambridge Isotope Laboratories Inc. (Andover, MA, USA): [d_10_]-L-isoleucine, [d_8_]-L-valine, [d_2_]-glycine, [d_3_]-methyl-L-methionine, [d_5_]-ring-L-phenylalanine, [d_5_]-L-glutamine, [d_5_]-indole-L-tryptophan, [d_4_]-L-lysine:2HCl, and [d_4_]-L-citulline.

### 2.4. Electrospray Ionisation Tandem Mass Spectrometric (ESI-MS/MS) Analysis of Acylcarnitines

The electrospray ionisation tandem mass spectrometry (ESI-MS/MS) method for determination of serum acylcarnitines as described by Vreken et al. [16] was adapted to determine acylcarnitines in urine. To a microcentrifuge tube, 10 *μ*L centrifuged urine was added to 400 *μ*L of the deuterated acylcarnitines (internal standard solution) with the following concentrations: 30.45 *μ*mol/L for [methyl-d_3_]-L-carnitine·HCl, 20.83 *μ*mol/L for [d_3_]acetyl-L-carnitine·HCl, 19.69 *μ*mol/L for [3,3,3-d_3_]propionyl-L-carnitine·HCl, 17.73 *μ*mol/L for [d_9_]isovaleryl-L-carnitine·HCl, 15.43 *μ*mol/L for [8,8,8-d_3_]octanoyl-L-carnitine·HCl, and 11.47 *μ*mol/L for [16,16,16-d_3_]hexadecanoyl-L-carnitine·HCl. The samples were then evaporated to dryness under a gentle stream of nitrogen at 55°C. To the dried residue, 200 *μ*L 3N butanolic HCl was added and the samples were incubated at 55°C for 20 min. The butylated samples were evaporated to dryness again under a stream of nitrogen at 55°C. The dried residue was reconstituted in water : acetonitrile (50 : 50) (v/v) containing 0.1% formic acid. 

An Agilent 1200 series liquid chromatograph (Santa Clara, CA, USA) with a 96-well plate sampler was used for sample handling as well as mobile phase delivery. Samples (10 *μ*L of each) were injected, and a constant flow rate of 0.2 mL/min was maintained throughout the run. The mobile phase consisted of 0.1% formic acid in water : acetonitrile (50 : 50) (v/v). The tandem mass spectrometry (MS/MS) analysis was performed on an Agilent 6410 Triple Quadrupole (Santa Clara, CA, USA) in positive ionisation. Acylcarnitines were analysed with a precursor ion scan, after controlled collision induced dissociation, with a fragmentor voltage of 135 V and collision energy of 20 V. All carnitine, acylcarnitine, and other butylated species that yielded a charged mass of 85 Da after fragmentation were detected. Acylcarnitines were quantified by comparison of the signal intensity of carnitine and acylcarnitines against the signal intensity of the corresponding deuterated analogues. The concentrations of analysed carnitine and acylcarnitines were expressed as mmol/mol creatinine.

### 2.5. Liquid Chromatography Tandem Mass Spectrometric (LC-MS/MS) Analysis of Acylcarnitine Isomers

The LC-MS/MS method for the separation and identification of short-chain acylcarnitine isomers as described by Ferrer et al. [17] was used, with minor modifications to separate butyrylcarnitine and isobutyrylcarnitine. A 100-*μ*L volume of urine was prepared the same as for the ESI-MS/MS method. High-performance liquid chromatography was performed on an Agilent 1200 series liquid chromatograph equipped with a Luna C18(2) column (150 mm × 2.00 mm, particle size 5 *μ*m) from Phenomenex (Torrance, CA, USA). Mobile phase A consisted of 10 mM ammonium acetate in water and mobile phase B of 10 mM ammonium acetate in methanol. Column temperature was maintained at 20°C and the flow rate at 0.2 mL/min. The samples (10 *μ*L) were injected and the mobile phase composition was changed from 40% of B to 60% of B over 15 min, after which the percentage of B was further increased to 100% over the next 5 min and kept for 5 min. The percentage of B was changed back to 40% over 3 min, and the column re-equilibrated for 7 min. 

The MS/MS analysis was performed on an Agilent 6410 Triple Quadrupole (Santa Clara, CA, USA) in positive ionisation after controlled collision induced dissociation, with optimised fragmentor voltages and collision energies for butyrylcarnitine, isobutyrylcarnitine, and the deuterated analogues used for quantification. Mass spectrometry conditions were optimised with the MassHunter optimiser software from Agilent. Acylcarnitines were analysed in multiple reaction monitoring (MRM) mode, with the following transitions being monitored, *m/z* 288 → 85 for both butyrylcarnitine and isobutyrylcarnitine, *m/z* 277 → 85 for [3,3,3-d_3_]propionyl-L-carnitine·HCl, *m/z* 311 → 85 for [d_9_]isovaleryl-L-carnitine·HCl and *m/z* 347 →to 85 for [8,8,8-d_3_]octanoyl-L-carnitine·HCl. The concentrations of C4-carnitine isomers were determined by comparing the signal intensity of acylcarnitines against the signal intensity of the corresponding deuterated analogues. For both butyrylcarnitine and isobutyrylcarnitine, a linear relationship between concentration and intensity existed, with *R* ^2^ > 0.99. The concentrations of analysed acylcarnitine isomers were expressed as mmol/mol creatinine.

### 2.6. Electrospray Ionisation Tandem Mass Spectrometric (ESI-MS/MS) Analysis of Amino Acids

Samples were prepared in the same way as for the analysis of acylcarnitines. Added internal standard solution contained deuterated amino acids with the following concentrations: 17.43 *μ*mol/L for [d_10_]-L-isoleucine, 32.20 *μ*mol/L for [d_8_]-L-valine, 15.99 *μ*mol/L for [d_2_]-glycine, 3.98 *μ*mol/L for [d_3_]-methyl-L-methionine, 5.77 *μ*mol/L for [d_5_]-ring-L-phenylalanine, 3.28 *μ*mol/L for [d_5_]-L-glutamine, 14.89 *μ*mol/L for [d_5_]-indole-L-tryptophan, 14.16 *μ*mol/L for [d_4_]-L-lysine:2HCl, and 4.21 *μ*mol/L for [d_4_]-L-citrulline. 

Amino acids were analysed in MRM mode for the following transitions: glycine *m/z* 132 → 30, [d_2_]-glycine *m/z* 134 → 32, alanine *m/z* 146 → 44, serine *m/z* 162 → 60, proline and arginine *m/z* 172 → 70, valine *m/z* 174 → 72, [d_8_]-L-valine, threonine *m/z* 176 → 74, leucine and isoleucine *m/z* 188 →86, [d_10_]-L-isoleucine *m/z* 191 →89, methionine *m/z* 206 → 104, [d_3_]-methyl-L-methionine *m/z* 209 → 107, histidine *m/z* 212 to 110, citrulline *m/z* 215 → 113, phenylalanine *m/z* 222 → 120, [d_5_]-ring-L-phenylalanine *m/z* 227 → 125, tyrosine *m/z* 238 → 136, aspartic acid *m/z* 246 → 144, glutamic acid *m/z* 260 → 158, glutamic acid-d_3_ *m/z* 263 → 161, tryptophan *m/z* 261 → 159, [d_5_]-indole-L-tryptophan *m/z* 266 → 164, lysine *m/z* 203 → 84, and [d_4_]-L-lysine: 2 HCl *m/z* 207 → 88. The concentrations of the amino acids were determined by comparing the signal intensity of the amino acids against the signal intensity of the corresponding deuterated analogues. The concentrations of analysed amino acids were expressed as mmol/mol creatinine.

### 2.7. Liquid Chromatography Tandem Mass Spectrometric (LC-MS/MS) Analysis of Branched-chain Amino Acids

Samples were prepared in the same manner as for the determination of acylcarnitine isomers. High-performance liquid chromatography was performed on an Agilent 1200 series liquid chromatograph equipped with a Luna C18(2) column (150 mm × 2.00 mm, particle size 5 *μ*m) from Phenomenex (Torrance, CA, USA). Column temperature was maintained at 20°C, and the flow rate at 0.2 mL/min. Mobile phase A consisted of 0.1% formic acid in water, and mobile phase B of 0.1% formic acid in methanol. The samples (10 *μ*L) were injected, and the mobile phase composition was changed from 40% of B to 60% of B over 15 min, after which the percentage of B was further increased to 100% over the next 5 min and kept for 5 min. The percentage of B was changed back to 40% over 3 min, and the column re-equilibrated for 4 min. The MS/MS analysis was performed on an Agilent 6410 Triple Quadrupole (Santa Clara, CA, USA) in positive ionisation after controlled collision induced dissociation with optimised fragmentor voltages and collision energies for leucine, isoleucine, valine, and the deuterated analogues used for quantification. Mass spectrometry conditions were optimised with the MassHunter optimiser software from Agilent. Branched-chain amino acids were analysed in MRM mode, for the same transitions as described in the ESI-MS/MS analysis of amino acids. The concentrations of the branched-chain amino acids were determined by comparing the signal intensity of the branched-chain amino acids against the signal intensity of the corresponding deuterated analogues. The concentrations of analysed branched-chain amino acids were expressed as mmol/mol creatinine.

### 2.8. Statistical Analysis

A paired *t*-test was used to demonstrate statistically significant differences between the test samples. In all cases, statistical significance was set at *P* < .05.

## 3. Results

### 3.1. Acylcarnitine Analysis (ESI-MS/MS)

A comparison between the acylcarnitine profiles in baseline urine samples of the first test group (*n* = 30) obtained before and after administration of a combination of acetylsalicylic acid and acetaminophen, revealed a statistically significant increase in the excretion of C3- (*P* = .05), C4- (*P* = .00), and C5-carnitine moieties (*P* = .00). Comparison of the acylcarnitine profiles in baseline urine samples of the second test group (*n* = 7) to the profiles obtained after the administration of a therapeutic dose of acetylsalicylic acid alone, revealed a statistically significant increase in the excretion of C4-carnitine (*P* = .03) (Table 1). However, a comparison between the acylcarnitine profiles obtained before and after the administration of a therapeutic dose of acetaminophen (*n* = 7), revealed a decreased excretion of various dicarboxylic acid carnitine conjugates, that is, C4-DC (*P* = .05), C5-DC/C10-OH-carnitine (*P* = .01), C6-DC (*P* = .01), and C8-DC (*P* = .00) (Table 1).

### 3.2. Acylcarnitine Isomer Analysis (LC-MS/MS)

**Figure 2:**
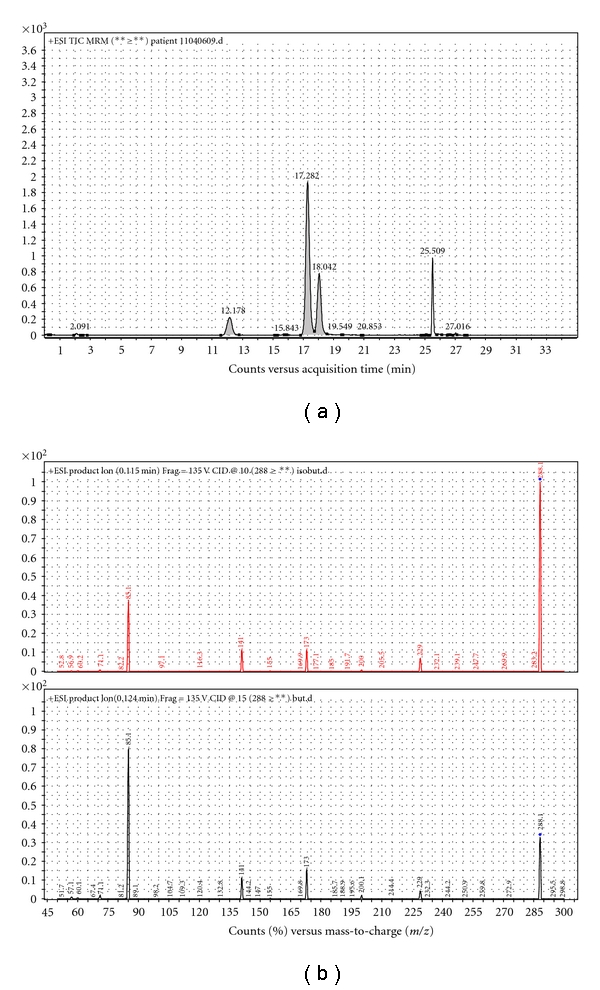
Separation and identification of short-chain acylcarnitine isomers. (a) In the LC-MS/MS analysis of acylcarnitine isomers, isobutyrylcarnitine is detected at 17.282 min and butyrylcarnitine at 18.042. (b) Product ion scan of *m/z* 288 for both isobutyrylcarnitine and butyrylcarnitine.

Comparing the LC-MS/MS baseline data with that obtained after the administration of a combination of acetylsalicylic acid and acetaminophen, revealed that the increase in the excretion of C4-carnitine was due to the increased excretion of isobutyrylcarnitine (*P* = .04) rather than due to the increase in butyrylcarnitine (*P* = .46) (Figure 2). The results obtained for the different C4-carnitine isomers revealed no statistically significant difference when either butyrylcarnitine or isobutyrylcarnitine concentrations in baseline urine samples were compared to samples (*n* = 7) taken after acetylsalicylic acid administration (*P* = .61 and *P* = .20) and samples (*n* = 7) taken after acetaminophen administration (*P* = .62 and *P* = .32).

### 3.3. Amino Acid Analysis (ESI-MS/MS & LC-MS/MS)

Concerning the amino acid analysis, a statistically significant decrease in the excretion of isoleucine (*P* = .00), leucine (*P* = .04), valine (*P* = .01), and tryptophan (*P* = .01) (Table 2) was evident in the urine samples obtained after acetylsalicylic acid and acetaminophen administration (*n* = 30). A comparison between the amino acid profiles in baseline urine samples and samples taken after acetylsalicylic acid (*n* = 7) and acetaminophen (*n* = 7) administration revealed a statistically significant decrease in the excretion of alanine, glycine, the branched-chain amino acids, methionine, phenylalanine, tyrosine, lysine, histidine, aspartic acid, and glutamic acid. In addition, significantly less proline/arginine, tryptophan, and serine were present in the urine after acetaminophen administration, but not after acetylsalicylic acid administration (Table 2).

## 4. Discussion

Acetylsalicylic acid and acetaminophen are generally used as probe substrates in the evaluation of biotransformation metabolism and oxidative stress status in humans [18]. Careful investigation of the acylcarnitine profiles of such subjects revealed the presence of increased concentrations of C3-, C4- and, C5-carnitine species in their urine. Since these acylcarnitine species reached concentrations normally associated with inborn errors of metabolism in some subjects [19], it was investigated whether this phenomenon originated from the administration of acetylsalicylic acid or acetaminophen, or a combination thereof. An increased excretion of various acylcarnitines derived from inhibited fatty acid oxidation, such as octanoylcarnitine and palmitoylcarnitine [7] as well as certain dicarboxylic acid carnitines with chain lengths ranging from C6 to C12 [10] were expected after acetylsalicylic acid administration. In the case of acetaminophen administration, an increased excretion of palmitoylcarnitine was expected [20]. However, our analyses revealed a statistically significant increase in the excretion of C4-carnitine in the case of acetylsalicylic acid administration, a statistically significant decrease in the excretion of various dicarboxylic acid carnitine conjugates after acetaminophen administration, and a statistically significant increase in the excretion of C3-, C4-, and C5-carnitine in the case of the combined administration. The most important observation in this respect is that the increased excretion in C4-carnitine was due to the intake of acetylsalicylic acid and not acetaminophen or a combined effect. Although there was no statistically significant difference in the C3- and C5-carnitine concentrations after acetylsalicylic acid administration (*n* = 7), as in the case of the combined administration, one cannot exclude that acetylsalicylic acid could be the cause of this increase, taking cognisance of the relatively small sample size.

It is known that butyrylcarnitine (C4-carnitine) accumulates with deficient or inhibited short-chain acyl-CoA dehydrogenase and that isobutyrylcarnitine (C4-carnitine) accumulates when isobutyryl-CoA dehydrogenase is deficient or inhibited [2]. In the case of C5-carnitine, the isomers include isovalerylcarnitine and 2-methylbutyrylcarnitine, which will accumulate with deficient or inhibited isovaleryl-CoA dehydrogenase and 2-methylbutyryl-CoA dehydrogenase [3] (Figure 1). It was demonstrated by Glasgow et al. [15] that the metabolites of acetylsalicylic acid can exercise mixed inhibition on *β*-oxidation at the level of the LCHAD activity of the MTE, as a result of structural similarities between acetylsalicylic acid and the acyl-part of fatty acids. It was, therefore, necessary to determine whether the observed increased excretion of C4-carnitine was due to increased butyrylcarnitine or isobutyrylcarnitine. The chromatographic separation of these isomers from each other in both the baseline samples and the samples obtained after acetylsalicylic acid and acetaminophen administration was of utmost importance and during the chromatographic separation, when performing a precursor ion scan for a product with an *m/z* of 85, two precursor ions were detected for C4-carnitine with an *m/z* of 288 (Figure 2). 

A comparison of the amount of the C4-carnitine isomers in the baseline samples to the test samples (after acetylsalicylic acid and acetaminophen administration, resp.) revealed no statistically significant difference. However, in the samples analysed to investigate a possible combined effect of acetylsalicylic acid and acetaminophen administration (*n* = 30) a statistically significant increase in only the isobutyrylcarnitine concentration was observed. These results support the hypothesis that acetylsalicylic acid may have an inhibitory effect on short-chain fatty acid metabolism, possibly at the level of isobutyryl-CoA dehydrogenase involved in the catabolism of branched-chain amino acids. Since there was no observed effect on these metabolic pathways after acetaminophen administration alone, the possibility also exists that the increase in C5-carnitine species observed after the combined administration may be due to the same inhibitory effect on the short-chain fatty acid metabolism. In this case, it can be at the level of the isovaleryl-CoA dehydrogenase and S-2-methylbutyryl-CoA dehydrogenase enzymes. Furthermore, it has been demonstrated that methyl-enecyclopropylacetic acid, a metabolite of hypoglycin can irreversibly inhibit all three acyl-CoA dehydrogenase enzymes in the branched-chain amino acid metabolism [21]. 

To further demonstrate the possible inhibitory effect of acetylsalicylic acid and/or its metabolites on the branched-chain amino acid metabolism, the total amino acid profiles were analysed. These analyses were also done on both baseline samples and samples taken after acetylsalicylic acid and acetaminophen administration, individually and combined. The comparison between the amino acid profiles in baseline urine samples and samples taken after acetylsalicylic acid (*n* = 7) and acetaminophen (*n* = 7) administration revealed a statistically significant decrease in the excretion of various amino acids, including the branched-chain amino acids. While the combined administration of acetylsalicylic acid and acetaminophen revealed a statistically significant decrease in only the branched-chain amino acids and tryptophan. However, an increase in branched-chain amino acid excretion after acetylsalicylic acid administration was expected due to the proposed inhibitory effect downstream in the branched-chain amino acid catabolism. 

In only some of the subjects, the amount of excreted isobutyrylcarnitine after the administration of acetylsalicylic acid, reached concentrations normally associated with isobutyryl-CoA dehydrogenase deficiency. In this regard, we would like to speculate as to the possible implications of this observation. Since not all individuals are affected to the same degree, it is possible that the inhibitory effect of acetylsalicylic acid is more pronounced in carriers of the isobutyryl-CoA dehydrogenase deficiency or in individuals with rate-limiting polymorphisms in the same enzyme system. If this is indeed the case, it opens the opportunity to investigate whether the administration of acetylsalicylic acid can be used to predict the carrier status in isobutyryl-CoA dehydrogenase deficiency. Since the first patient with isobutyryl-CoA dehydrogenase deficiency was diagnosed only over a decade ago [21], and since there is substantial variation in the clinical presentation of this deficiency, it also poses the opportunity to investigate other biochemical effects involved in the pathology of deficient isobutyryl-CoA dehydrogenase, as the elucidation of the development of phenotypic characteristics of metabolic diseases remains a formidable challenge.

In conclusion, from the literature sources, it is clear that acetylsalicylic acid and/or its metabolites have various effects on metabolism and, especially, on mitochondrial function. In this study, the first *in vivo* evidence, in human subjects, of a statistically significant increase in isobutyrylcarnitine excretion as a result of the administration of a therapeutic dose of acetylsalicylic acid was provided. Since it was previously demonstrated that the structural similarities between acetylsalicylic acid and the acyl-portion of fatty acids can result in mixed inhibition on *β*-oxidation [15], we propose a possible inhibitory effect on isobutyryl-CoA dehydrogenase, as elevated isobutyrylcarnitine excretion is generally a result of a deficiency in the branched-chain amino acid catabolism.

## Figures and Tables

**Table 1:** Paired *t*-test values (*P*-values) of acylcarnitine species in baseline urine samples compared to acylcarnitine species in urine samples obtained after acetylsalicylic acid administration (*n* = 7), acetaminophen administration (*n* = 7), and combined administration of both acetylsalicylic acid and acetaminophen (*n* = 30).

Acylcarnitine species	Paired *t*-test (*P*-value)		
	Acetylsalicylic acid administration (*n* = 7)	Acetaminophen administration (*n* = 7)	Combined administration (*n* = 30)
C0-carnitine	0.18	0.20	0.72
C2-carnitine	0.08	0.06	0.87
C3-carnitine	0.96	0.88	0.05*
C4-carnitine	0.03*	0.31	0.00*
C4-OH-carnitine	0.18	0.12	0.32
C4-DC-carnitine	0.16	0.05*	0.08
C5-carnitine	0.73	0.85	0.00*
C5-OH-carnitine	0.69	0.33	0.35
C5-DC/C10-OH-carnitine	0.17	0.01*	0.26
C5 : 1-carnitine	0.24	0.50	0.29
C6-carnitine	0.20	0.62	0.98
C6-DC-carnitine	0.29	0.01*	0.77
C8-carnitine	0.41	0.10	0.20
C8-DC-carnitine	0.84	0.00*	0.55
C10-carnitine	0.20	0.11	0.20
C5 : 1-DC/C10 : 1-OH-carnitine	0.82	0.10	0.90
C12-carnitine	0.20	0.11	0.15
C14-carnitine	0.41	0.20	0.70
C16-carnitine	0.21	0.07	0.95

*Differences are considered to be statistically significant compared to baseline values if *P* < .05.

**Table 2:** Paired *t*-test values (*P*-values) of amino acids in baseline urine samples compared to amino acids in urine samples obtained after acetylsalicylic acid administration (*n* = 7), acetaminophen administration (*n* = 7), and combined administration of both acetylsalicylic acid and acetaminophen (*n* = 30).

Amino Acids	Paired *t*-test (*P*-value)		
	Acetylsalicylic acid administration (*n* = 7)	Acetaminophen administration (*n* = 7)	Combined administration (*n* = 30)
Alanine	0.00*	0.00*	0.50
Glycine	0.03*	0.02*	0.82
Valine	0.02*	0.01*	0.01*
Leucine	0.04*	0.03*	0.04*
Isoleucine	0.02*	0.01*	0.00*
Methionine	0.01*	0.01*	0.73
Proline/Arginine	0.06	0.02*	0.21
Phenylalanine	0.02*	0.01*	0.43
Tryptophan	0.12	0.03*	0.01*
Serine	0.06	0.03*	0.32
Threonine	0.19	0.07	0.31
Tyrosine	0.01*	0.00*	0.13
Lysine	0.03*	0.03*	0.45
Histidine	0.01*	0.00*	0.20
Aspartic acid	0.05*	0.05*	1.00
Glutamic acid	0.02*	0.02*	0.39

*Differences are considered to be statistically significant compared to baseline values if *P* < .05.
